# A framework for planning organ at risk volume margin derivation for the small bowel during magnetic resonance-guided radiotherapy

**DOI:** 10.1016/j.phro.2026.101036

**Published:** 2026-07-13

**Authors:** Saskia L.C. Damen, Astrid L.H.M.W. van Lier, Cornel Zachiu, Martijn P.W. Intven, Bas W. Raaymakers

**Affiliations:** Radiotherapy department, UMC Utrecht, Heidelberglaan 100 3584 CX Utrecht, Netherlands

**Keywords:** MR-guided radiotherapy, PRV margins, Small bowel

## Abstract

**Background and Purpose:**

The small bowel is highly radiosensitive and prone to motion during abdominal and pelvic radiotherapy, risking overdosage. This study extended a previously presented small bowel tracking framework to enable automated planning organ at risk volume (PRV) margin derivation.

**Materials and Methods:**

MR scans were obtained in 18 patients treated for abdominal lesions, with abdominal compression. The mean motion, maximum displacement and position of the small bowel during the acquisition (7.5 min) were determined. The position of the small bowel over time was used to derive PRV margins based on geometrical coverage.

**Results:**

Displacements >20 mm in the craniocaudal direction were observed, with approximately half of the bowel voxels experiencing positional displacements close to 10 mm. Despite this substantial motion, margin analysis demonstrated that applying a 4.5 mm isotropic margin around the delineated bowel loops was sufficient to cover 95% of the bowel voxels occupied for at least 5% of the time in 90% of the cases. It was shown that shorter acquisitions (2.5 min) already gave stable margin results, supporting the feasibility of using individualized PRV margins to balance motion coverage with treatment planning flexibility.

**Conclusions:**

While the small bowel can undergo considerable intrafraction displacements during treatment, most of this motion can be effectively encompassed using a moderate intrafraction PRV margin for this specific cohort. This study presents an automated framework to derive patient- and treatment-specific margins, directly from bowel tracking data, enabling tailored coverage-based margins.

## Highlights

• Individual bowel loops reduced organ-at-risk volume compared to the bowel bag.
• Patient- and treatment-specific margins were derived directly from bowel tracking.
• Small bowel displacements >20 mm in craniocaudal direction were observed.
• A 4.5-mm isotropic margin covered 95% of bowel motion.
• Margin results remained stable with shorter acquisitions (i.e., 2.5 min.).

## Graphical abstract

**Figure:**
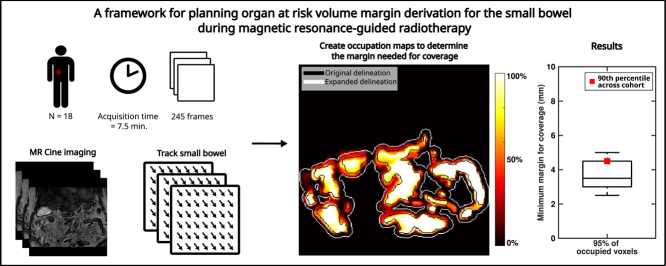

## 1 Introduction

The introduction of the Magnetic Resonance Linear Accelerator (MR-Linac) brought the excellent soft-tissue contrast of the MR into the treatment room, improving daily imaging of the tumor sites, which is especially beneficial in areas such as the pelvis and abdomen [1]. This daily imaging opened the door to adaptive treatments, aimed at increasing accuracy of the dose to the target and sparing of the surrounding organs-at-risk (OARs) with reduced margins [2], [3]. This has the potential to enable, for example, hypo-fractionated treatments, in which the dose to the target is increased and the number of fractions is reduced [4], [5]. An increase in dose delivery, however, also means an increase in delivery time thus a bigger impact of intrafraction motion.

An important OAR for abdominal and pelvic treatments is the small bowel. Common side effects for this OAR include radiation enteritis, ulceration, and bowel obstruction, occurring in a considerable percentage of patients [6], [7], [8], [9]. The bowel is often located close to the target volumes in both pelvic and abdominal radiotherapy, and can limit the achievable dose to the target [10]. This is especially the case in hypo-fractionated treatments or stereotactic ablative body radiotherapy [11]. In current treatment planning the bowel is often delineated as the full bowel bag. Although this includes the volume where the bowel loops can potentially be, it therefore also includes parts where no bowel is located at that point in time. There is an expected benefit in delineating individual bowel loops rather than the entire bowel bag, since a reduction in volume might lead to more flexibility in treatment planning and better predict side effects [12], [13]. However, to make this possible, first some challenges must be overcome for the imaging and tracking of the small bowel loops.

To get a good insight into the motion of the small bowels, the individual bowel loops need to be visible over time. During MR-guided radiotherapy it is possible to track organs during treatment. However, imaging of the small bowel remains challenging due to its mobility and the slow speed of 3D MR imaging, when acquiring with adequate contrast and resolution to image individual bowel loops. These challenges are often solved with the use of contrast agents to distend the bowel [14], [15] and breath-holds to reduce respiratory motion blurring [15], [16]. Besides the bowel distention not being favorable for the anatomy during radiotherapy, the repeated use of oral contrast agents can become an issue over the course of treatment and is not always stable for prolonged times [17], which is also the case for breath-holds. Therefore, in previous work an MRI-based method was developed to image and track the small bowel over a period of time on the MR-linac [18]. By tracking the small bowel, the motion over time can be obtained and analyzed.

Motion and displacement of the small bowel can be divided into two categories: periodic motion, caused by peristalsis and respiration, and non-periodic motion, for example due to passing chyme in the intestine and filling of the bladder [19], [20]. Regular peristalsis has amplitudes around 6.65 mm [20], whereas the second category can have motion amplitudes which can go up to the range of centimeters [16]. These unexpected displacements can lead to unwanted increased radiation exposure to the small bowel. To take this mobility into account in treatment planning, for example Planning Organ-at-risk Volume (PRV) margins can be applied.

The PRV margin concept was introduced in ICRU report 62 to account for organ motion and uncertainties [21]. McKenzie et al. proposed a method for deriving PRV margins that ensures OAR dose is not underestimated in 90% of patients [22]. Since for serial organs, such as the bowel, the maximum dose is the most important limitation, it is important that the organ lies within the area it is expected to be in. In practice this leads to different usages, such as a similar definition as that of planning target volume (PTV) margins [22] or looking at geometric coverage for interfraction motion [23], [24], [25]. Hysing et al. determined PRV margins for interfraction bowel delineations by calculating the geometric coverage on repeat MR imaging. This led to relatively large margins, 5–10 mm to encompass 85%–95% of volumes having an occupancy of ≤75%, including a large volume unoccupied by bowel in each fraction. Patient-specific analysis reduced this margin but required weekly repeat computed tomography (CT) scans and manual delineations, to determine interfraction coverage. Uchinami et al. also derived margins from 3 CT scans acquired during the planning phase and found interfraction margins of 14.0 mm for the small intestine. With MR-guided radiotherapy, the bowel can be re-delineated each fraction, meaning only intrafraction motion needs to be accounted for, potentially allowing smaller PRV margins.

The aim of this paper was to adapt the established concept of geometric coverage for PRV margin derivation to automatically generate occupancy maps from cine MR imaging on the MR-Linac, enabling intrafraction motion coverage assessment for the small bowel. The method was demonstrated in a cohort of patients, treated on lesions in the upper abdominal area. Margins, robustness and volume reduction with respect to full bowel bag delineations were determined. Furthermore, additional motion statistics were derived to get a better insight into the motion present and its potential impact on more complex radiotherapy treatments.

## 2 Materials and methods

### 2.1 Data acquisition

The dataset consisted of all patients treated for upper abdominal tumors between February and October 2024 in our institution, who provided informed consent under the MOMENTUM study (NCT04075305), yielding a cohort of 20 patients (11 male, 9 female) with a median age of 68 years (range 33–81 years). All subjects were scanned on a 1.5 T MRL (Unity, Elekta AB), during the planning phase of the online treatment, with the use of abdominal compression bands (Aspen® Lumbar support), as described by the treatment protocol in our department. Patients with a low visceral fat percentage (visc. fat-%≤ 15%) were excluded from the dataset, due to poor bowel loop visibility [18]. This was the case for 2 patients, resulting in a dataset of 18 patients (10 male, 8 female), median age 68 (33–81) years.

MRI scans were acquired for 7.5 min (1.8 s/scan) continuously, with the cine scan described by Damen et al. [18]. The total acquisition time was consistent with the recommended acquisition time by Barten et al. [26]. The fifth frame was selected as the reference scan, resulting in a total of 246 frames for analysis. The small bowel loops were delineated manually in MIM${}^{\text{TM}}$ (MIM Software, Cleveland, OH, USA) on the reference scan and approved by an expert radiation oncologist. The resulting delineations allowed restricting the motion analysis exclusively to the small bowel, rather than the entire bowel bag.

Small bowel displacements and deformations were obtained by means of an optical flow-based deformable image registration algorithm [27]. In previous work the precision of the algorithm was verified [18]. A further expansion of the accuracy using simulations is performed (Fig. S1) and can be found in the supplementary material.

### 2.2 Motion analysis

The registration algorithm returns Deformation Vector Fields (DVFs) in 3D, containing all three orthogonal components of the vectors for all frames relative to the reference frame. This means that spatiotemporal information is available for the small bowel motion of each subject. To describe the motion present, different metrics were extracted, which are listed below.

First, the mean motion of the bowel loops per frame was investigated. In this context motion is defined as the absolute vector length per orthogonal direction, of which the mean value is calculated.

Second, the 95% peak-to-peak motion (2.5%–97.5%) of each voxel in the reference image is taken, as a noise-robust surrogate for the maximum positional difference. For each subject a Motion-Volume Histogram (MVH) was generated, indicating the percentage of bowel voxels experiencing certain amounts of motion amplitudes. From these MVHs, the motion amplitude experienced by at least 90% (M90%), 50% (M50%) and 10% (M10%) of the voxels in the volume are extracted.

### 2.3 Occupancy maps and PRV margins

For an estimation of the PRV margins, a similar method to that of Hysing et al. [23] was used. The bowel loop masks were deformed based on the obtained DVFs, giving the location of the small bowel loops over time. By summing all deformed masks and normalizing the result between, an occupancy map was generated, indicating for each voxel the fraction of time it was occupied by small bowel.

Next, the original bowel mask was systematically expanded by varying margins. For each expansion, the number of voxels with a specified occupancy level that fell outside the original mask was quantified. This allowed for the identification of the minimum margin required to encompass at least x% of the voxels that were occupied more than y% of the time. All motion was defined as all voxels that were occupied for at least 5% of the time, to filter out noisy voxels. The margin was defined as the 90th percentile value across the cohort, as defined by McKenzie et al. [22]. Margins were calculated for different coverages and occupancies.

The PRV volumes were compared to the complete bowel bag volumes, as the (expanded) bowel bag-to-bowel loop ratio (BLR), to estimate the sparing of volume using PRV margins as compared to the bowel bag delineations.

(1) $$\text{BLR}=\frac{V_{bowelbag}}{V_{bowelloops}+V_{margins}}$$

To assess the stability of the margin estimation framework, analyses were performed on temporal subsets of the full acquisition. Margins were calculated using data segments of 1.5 min (50 frames), 3 min (100 frames), 4.5 min (150 frames), and 6 min (200 frames), and compared to those derived from the complete 7.5-minute (245 frames) dataset. A sliding window approach was employed to compute margins at multiple time points, enabling an evaluation of temporal consistency. For each subset, margins were determined based on 95% coverage of occupancy >5%. The inter-quartile range (IQR) of the resulting distribution was analyzed, as well as the difference of the median with the margin as determined by the full dataset.

## 3 Results

The accuracy analysis (Fig. S2), showed accuracies below voxel size (mean end-point error (EE) = 0.2–1.7).

**Fig. 1:**
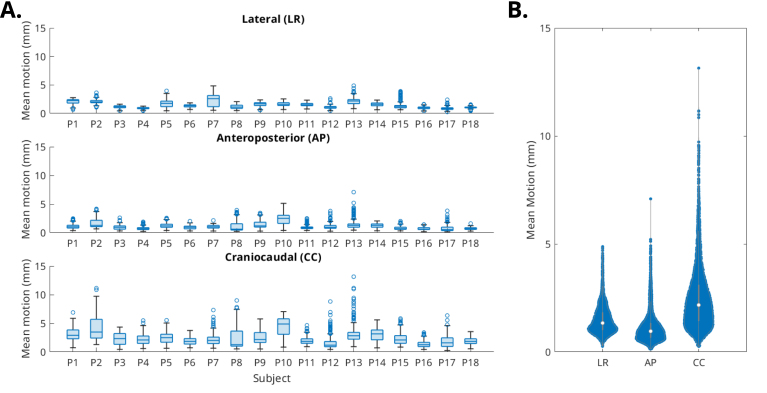
Mean motion for the different subjects at all time points during 7.5 min. A. The boxplots show the mean motion per frame for the different patients. B. The combined boxplot shows the mean motion over all time points for all patients combined, illustrating a larger motion component in craniocaudal direction.

Large inter-patient variability was present in the motion amplitude for all different orthogonal directions (Fig. 1A), with some patients showing a mean amplitude ⪆10 mm at some time points, whereas others only had a mean motion ≤5 mm. Overall, the motion of the small bowel was predominantly in the craniocaudal direction (Fig. 1B).

**Fig. 2:**
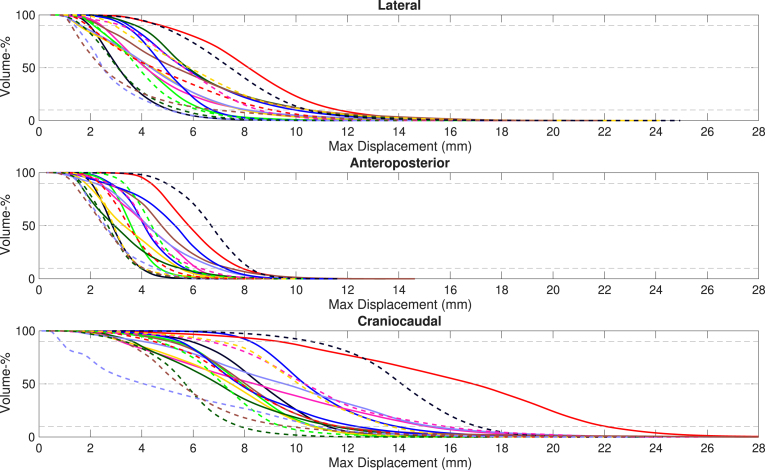
Largest positional difference for each patient combined in a motion-volume histogram. It shows the amount of displacement for each volume-% for each subject. The M10%, M50% and M90% are indicated by the horizontal dashed lines.

The MVHs of the largest positional differences in all three directions, showed the differences between patients (Fig. 2), especially in the craniocaudal direction. This was further highlighted by the M90%, M50% and M10% values, summarized in Table 1. For n = 17/18 M50% was ≥5 mm, and for n = 5/18 > 10 mm. For M10%, a value > 20 mm was found for one case, with the average over all patients 12.8 mm.

**Table 1:** Metrics associated with the motion volume histograms (MVHs), with the mean value over the cohort and the range of values, given in mm. M90%, M50% and M10% represent the minimum amount of maximum displacement for 90%, 50% and 10% of the volume.

	M90% (mm)		M50% (mm)		M10% (mm)	
	Mean	Range	Mean	Range	Mean	Range
Lateral	2.5	1.2–4.7	4.7	2.4–8.1	8.2	5.0–11.6
Anteroposterior	2.5	1.3–4.9	3.9	2.5–6.7	5.7	3.9–8.2
Craniocaudal	5.2	0.8–10.6	8.7	4.0–16.8	12.8	7.8–21.9

**Fig. 3:**
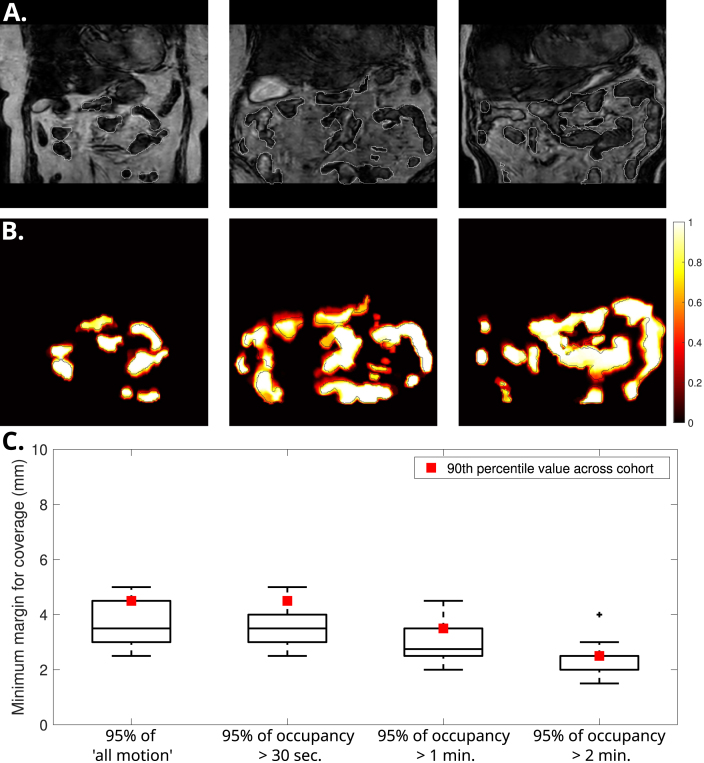
A. Slice of the reference frame of three examples, with the bowel delineation shown in white. B Examples of the occupancy maps of the same slice of the three patients as in A., including the delineation on the reference frame in black. C. Margins necessary per subject to achieve different amounts of coverage. The 90th percentile across the cohort is plotted as red squares.

The differences between patients could also be observed from the examples of occupancy maps (Fig. 3B), with the middle and right example extending further outside the delineation than the left example. This inter-patient variation further confirmed by the necessary margin needed for each subject to achieve a minimal coverage for a predefined time duration, in Fig. 3C. There was a variation between patients of 2.5 mm for the highest level of coverage (all motion), which decreased when the occupancy level increased. To cover all motion in 90% of the patients, an isotropic PRV margin of 4.5 mm was needed. This PRV margin reduced to 2.5 mm if the coverage was taken for voxels occupied >2 min.

**Fig. 4:**
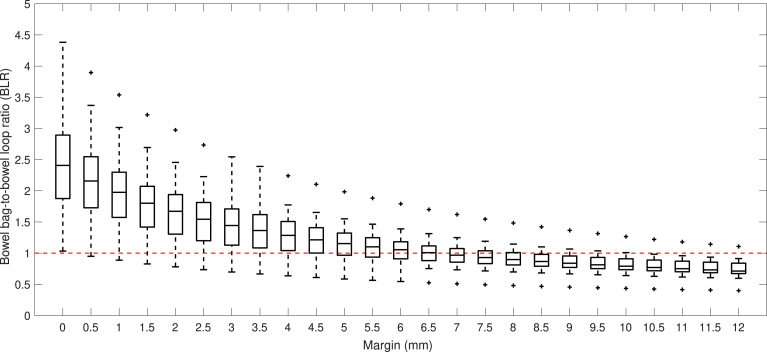
Differences in volume between the bowel loop delineation including margins and the delineation of the bowel bag for all patients. The dotted line shows the point where the bowel bag and bowel loop delineation are equal in volume.

The ratio between the bowel bag delineation and the bowel loop delineations expanded by varying margins, showed that up to a margin of 6.0 mm the median ratio was larger than 1 (Fig. 4), highlighting the trade-off between margin size and total volume. Furthermore, it was observed that with a margin of 4.5 mm, the PRV volume was around 20% smaller, compared to delineating the full bowel bag.

**Fig. 5:**
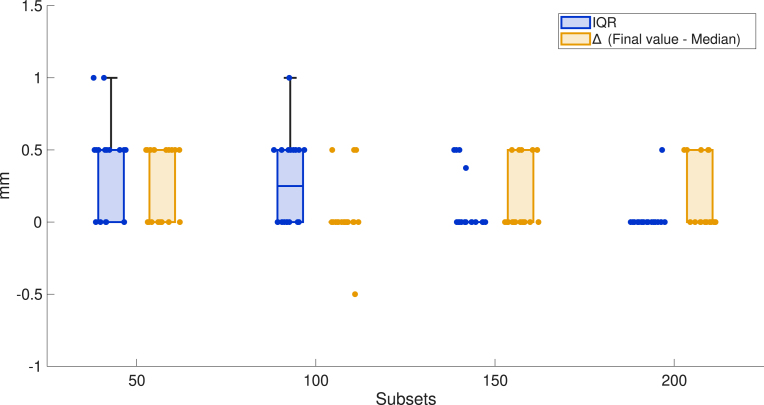
Stability of the derived margins as determined by subsets of different size at different time points for all subjects. The boxplot gives an overview of the IQR and the difference with the final margin for each patient.

The median margin for the 95% coverage varied between 0 and 0.5 mm as determined by different subsets (Fig. 5), indicating a good estimate of the final value, even for smaller subsets. The IQR of individual patients was between 0 and 0.5 mm for most patients at the smallest subsets and decreased further for larger subsets. The difference of the median with the final margin was between 0 and 0.5 for the coverage of 95%.

## 4 Discussion

In this study, small bowel motion during MR-guided radiotherapy was analyzed in a cohort of 18 patients for 7.5 min. Multiple metrics were employed to characterize motion over a 7.5-minute acquisition period. The accuracy of the tracking method (Fig. S2) was in line with recommendations [18], [28]. Displacements over 20 mm in the craniocaudal direction were observed, with approximately 50% of the voxels experiencing positional variations close to 10 mm over 7.5 min. Despite this substantial motion, margin analysis demonstrated that applying a 4.5 mm isotropic margin around delineated bowel loops was sufficient to cover 95% of the voxels occupied for at least 5% of the time in 90% of the cases.

Since average motion may not fully capture clinically relevant displacements, particularly if the motion is periodic and therefore not expected to significantly affect dose distribution due to location, the maximum positional difference per voxel was also assessed. These metrics underscored the extent of small bowel mobility during treatment, mainly in the craniocaudal direction, and the differences between subjects.

To calculate what margins would cover typical motion patterns in patients, a previously presented method [23] to determine interfraction PRV margins was adapted to intrafraction motion and automated. The found margin of 4.5 mm, to cover all voxels occupied by bowel for more than 5% in 90% of the patients, was smaller than values found in previous literature [23], [24], [25], while having a higher coverage. Although the maximum motion of the bowel loops was relatively large, a limited expansion of the delineation provides sufficient coverage. This can be attributed to several factors. First, large displacements may be of short duration, resulting in low occupancy over time. Second, the bowel loops often lie in close proximity to one another, so even a modest expansion can lead to overlap between delineations, effectively covering a broader area. The 90th percentile adopted from McKenzie et al. [22], assumes all motion deviations from the starting position could increase the delivered dose. While conservative, this ensures that the actual bowel dose is not underestimated.

The volume analysis, Fig. 4, highlighted the potential advantages of delineating individual small bowel loops with margin expansion. In reality anatomical boundaries might limit expansion, such as the posterior wall or surrounding organs. Although not applied here, such constraints could be incorporated depending on the intended application and may further amplify the observed effects. Up to a 5 mm margin, the resulting volumes were up to 50% smaller compared to the entire bowel bag and thus expected to offer greater flexibility in treatment planning. A drawback is the increased contouring time, although automatic segmentation solutions could decrease this time [29], [30], [31]. Furthermore, the proximity of bowel loops to the target is expected to influence its clinical relevance. In practice, only loops situated close to the high-dose region would need to be delineated, as is currently already done in OARs delineations for SBRT [32], [33]. This delineation could be used for the PRV framework. Absolute volume dose constraints, for (near-)maximum doses, can be applied to the total PRV structure, to avoid the effects of volume expansion on relative volume constraints [34]. This approach finds a balance between using the full bowel bag, being a strict way of constraining, and delineating single bowel loops without margin, which might be too tolerant. Its benefit is also expected to be closely related to the visceral fat percentage of the patient, where lower percentages would decrease the difference between bowel loops and the full bowel bag.

Analyzing temporal subsets of the full time series at various time points, the stability of the margin estimation method was assessed. Barten et al. [26] already showed the precision of bowel motion estimation and concluded that 7.5-minute MR acquisitions provide a reliable representation of motion patterns. Although the full on-table time is longer, the sequence could be acquired during the delineation phase. This would minimize the delay between image acquisition and the relevant treatment window, as Daamen et al. reported a median planning and beam-on time of 5 min. and 11 min. [33]. Here it is shown that, when aiming to cover 95% of the motion, the required margin remained relatively consistent across subsets, with only minor variations observed when using smaller subsets. Therefore, using a relatively short acquisition, e.g. 2.5 min, before treatment, could already yield consistent results across individual cases and allows for reliable margin estimation. These findings support considering an individual intrafraction margin strategy, in which bowel motion is assessed using a brief scan obtained immediately before irradiation.

This study included a relatively small cohort of patients with upper abdominal lesions scanned under abdominal compression, limiting the generalization of the findings to broader patient populations. Although the effect of abdominal compression on respiratory and tumor motion has been investigated, its effect on bowel motion remains less studied [35], [36]. Furthermore, location dependent variations in bowel motion could be further studied for radiotherapy purposes. Additionally, margin requirements depend on factors such as dose rate, and total treatment duration. In these patients mostly periodic motion was observed, whereas spontaneous events can still occur (not observed in this cohort), such as the passing of an air bubble. The proposed framework could be extended to detect such events and identify when they occur.

Despite these limitations, the methodology presented here offers a flexible framework that can be applied to other cohorts, such as patients with lesions in the lower abdomen, those without abdominal compression, or cases involving longer acquisition times, to further investigate more tailored margins, such as anisotropic margins or margins that adapt to the local dose gradient, for different groups. The framework could also be applied to other 3D cine MR sequences, which may enable its use in patient groups with lower visceral fat percentages, provided that a suitable MR sequence is found. Furthermore, another option would be to investigate the possibility of creating subject-specific margins, by acquiring data before the fraction delivery to determine the mobility and the necessary margins for the day.

## CRediT authorship contribution statement

**Saskia L.C. Damen:** Writing – original draft, Visualization, Validation, Project administration, Methodology, Investigation, Formal analysis, Data curation, Conceptualization. **Astrid L.H.M.W. van Lier:** Writing – review & editing, Supervision, Project administration, Methodology, Funding acquisition, Conceptualization. **Cornel Zachiu:** Writing – review & editing, Supervision, Software, Project administration, Methodology, Funding acquisition, Conceptualization. **Martijn P.W. Intven:** Writing – review & editing, Resources, Data curation. **Bas W. Raaymakers:** Writing – review & editing, Supervision, Methodology, Funding acquisition, Conceptualization.

## Declaration of competing interest

The authors declare the following financial interests/personal relationships which may be considered as potential competing interests: This work was supported by the Topconsortia for Knowledge and Innovation-Life Sciences and Health (AMPLIFY Project). Our department has a research agreement with Elekta AB. Elekta AB had no role in the preparation, review or approval of the manuscript and the decision to submit the manuscript.

## Appendix A Supplementary data

MMC S1 Accuracy analysis of the bowel tracking method. MMC S1
