# Supplementary material for: A framework for planning organ at risk volume margin derivation for the small bowel during magnetic resonance-guided radiotherapy
Source: Phys Imaging Radiat Oncol. 2026 Jul 13;40:101036. doi: 10.1016/j.phro.2026.101036 (PMC13375926; doi:10.1016/j.phro.2026.101036)
Supplement: MMC S1 — Accuracy analysis of the bowel tracking method. [file mmc1.pdf]

Supplementary Material

Evaluation of tracking accuracy

In Damen et al. 2025 the optical flow algorithm for small bowel tracking was validated using the structural similarity index (SSIM) and inverse consistency (IC), measuring the similarity of the registered frames and the consistency of the registration respectively [1]. The IC and SSIM values were in line with current recommendations for the use of deformable image registration in radiotherapy. However, because of the lack of a ground truth, a measure of the accuracy was not available. To create a set of known deformations on the dataset, to evaluate the accuracy, a set of biomechanical simulations was designed. This accuracy analysis was used to support the observations and conclusions provided by the current work.

Three patients were randomly picked from the dataset presented in the study to perform the simulations on. The small bowel delineations were used to create a shell of the inner body excluding the small bowel (Fig. S.1). In FEBiostudio a finite element model was then setup, simulating the respiration with a fixed displacement of the cranial surface of the shell and a peristalsis like motion, created with a periodic pressure in the inner parts of the shell [2].
The resulting model was then extracted as nodes at the different time points and converted into deformation vector fields, which were applied on the reference scan, simulating motion. Gaussian noise with a variance of 0.0002 was added to the simulated frames. Furthermore, every fourth frame was blurred with a 4-pixel wide motion blur filter in the craniocaudal direction.
This new simulated scan collection was then registered back to the reference frame, using the optical flow algorithm employed here, and multiple metrics were calculated. The mean motion of the bowel loops was calculated, and the end point error (EE) was calculated. The EE is calculated as the euclidean distance between the vectors of the known deformations and the vectors determined by the registration algorithm.

$$EE = \sqrt{(x_{i,GT} - x_{i,REG})^2 + (y_{i,GT} - y_{i,REG})^2 + (z_{i,GT} - z_{i,REG})^2} \tag{1}$$

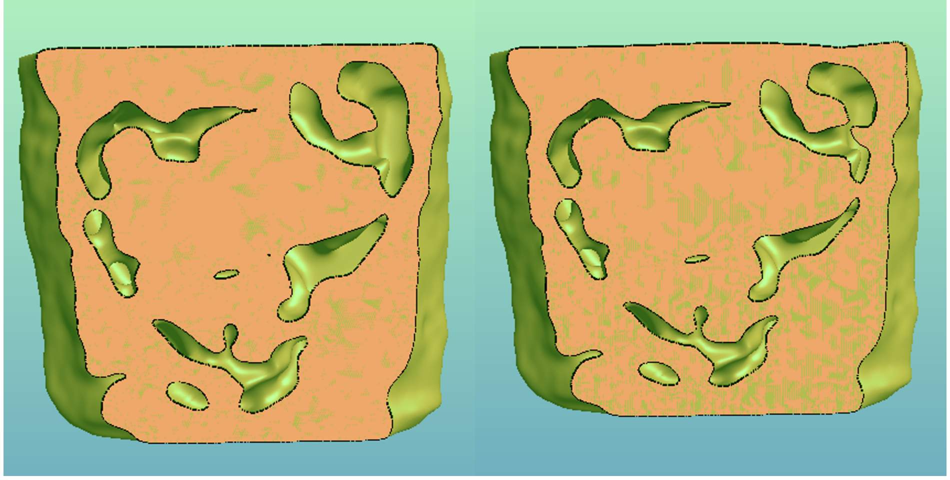

Figure S.1: Snapshots of the body shell at the starting time point and a later time point during which the body is compressed by respiration and the bowels are further deformed by a second pressure wave.

The mean EE was below 1 mm for most frames of P8 and P14, and slightly higher but below 2 mm for P1 (Fig. S.2).

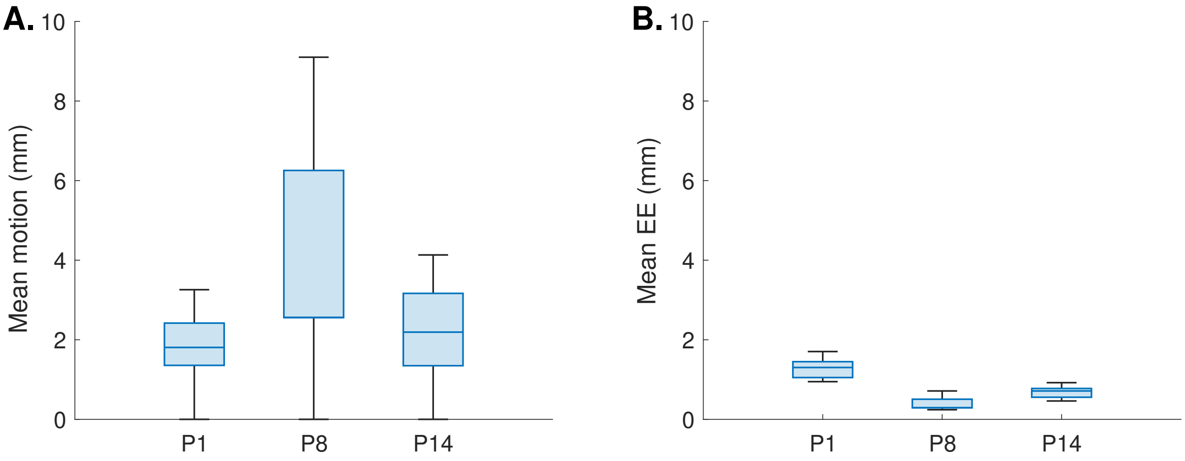

Figure S.2: Results of the simulations. A. shows the mean motion of the simulation of each frame. B. shows the end-point error (EE).

The mean motion is in the same range as the acquired cine scans for the patients, which is consistent with prior results on clinical data, see Fig. ??. The endpoint error was low, with means between 1 and 2 mm in all cases, indicating a high accuracy with respect to the simulated ground truth.
The simulations are not a direct accuracy measure of the acquired cine scans. These obtain more transient image artifacts which can not be simulated. However, the motion is approximated in such a way that they are a good indication that the optical flow algorithm manages to resolve the motion accurately.
The simulated motion was resolved with a mean end point error below 1.5 mm in most cases, indicating an accurate registration.

References

[1] S L C Damen, A L H M W Van Lier, C Zachiu, and B W Raaymakers. Bowel tracking for MR-guided radiotherapy: simultaneous optimization of small bowel imaging and tracking. *Physics in Medicine & Biology*, 70(7):075001, April 2025. ISSN 0031-9155, 1361-6560. doi: 10.1088/1361-6560/adbbac. URL https://iopscience.iop.org/article/10.1088/1361-6560/adbbac.

[2] Steve A. Maas, Benjamin J. Ellis, Gerard A. Ateshian, and Jeffrey A. Weiss. Febio: Finite elements for biomechanics. *Journal of Biomechanical Engineering*, 134(1):011005, 02 2012. ISSN 0148-0731. doi: 10.1115/1.4005694. URL https://doi.org/10.1115/1.4005694.
